# Cancer Incidence among Former Love Canal Residents

**DOI:** 10.1289/ehp.0800153

**Published:** 2009-05-05

**Authors:** Lenore J. Gensburg, Cristian Pantea, Christine Kielb, Edward Fitzgerald, Alice Stark, Nancy Kim

**Affiliations:** 1 University at Albany, State University of New York, Albany, New York, USA; 2 New York State Department of Health, Troy, New York, USA

**Keywords:** cancer, community health, exposure assessment, hazardous waste sites, Love Canal

## Abstract

**Background:**

The Love Canal was a rectangular 16-acre, 10-ft-deep chemical waste landfill situated in a residential neighborhood in Niagara Falls, New York. This seriously contaminated site came to public attention in 1978. Only one prior study examined cancer incidence in former residents of the Love Canal neighborhood (LC).

**Objective:**

In this study we aimed to describe cancer incidence in former LC residents from 1979 to 1996 and to investigate whether it differs from that of New York State (NYS) and Niagara County (NC).

**Methods:**

From 1978 to 1982, we interviewed 6,181 former residents, and 5,052 were eligible to be included in this study. In 1996, we identified 304 cancer diagnoses in this cohort using the NYS Cancer Registry. We compared LC cancer incidence with that of NYS and NC using standardized incidence ratios (SIRs), and we compared risks within the LC group by potential exposure to the landfill using survival analysis.

**Results:**

SIRs were elevated for cancers of the bladder [SIR_NYS_ = 1.44; 95% confidence interval (CI), 0.91–2.16] and kidney (SIR_NYS_ = 1.48; 95% CI, 0.76–2.58). Although CIs included 1.00, other studies have linked these cancers to chemicals similar to those found at Love Canal. We also found higher rates of bladder cancer among residents exposed as children, based on two cases.

**Conclusions:**

In explaining these excess risks, the role of exposure to the landfill is unclear given such limitations as a relatively small and incomplete study cohort, imprecise exposure measurements, and the exclusion of cancers diagnosed before 1979. Given the relatively young age of the cohort, further surveillance is warranted.

The Love Canal was a rectangular 16-acre, 10-ft-deep chemical waste landfill situated in a residential neighborhood in the city of Niagara Falls, in northwestern New York State (NYS). The trench was originally dug in 1894 by William T. Love to connect the upper and lower Niagara Rivers, thereby providing cheap hydroelectric power. The landfill was one of the most seriously contaminated hazardous waste sites in the United States, with approximately 21,800 tons of at least 200 different chemicals disposed by Hooker Chemical from 1942 to 1953 [New York State Department of Health (NYSDOH) 1981]. According to company records, these chemicals were predominantly hexachlorocyclohexanes (e.g., lindane), benzyl chlorides, organic sulfur compounds (e.g., lauryl mercaptans), chlorobenzenes, and sodium sulfide/sulfhydrates.

Before 1976, chemical odors, minor explosions, and fires were reported (Kim et al. 1982; NYSDOH 1981). In 1976–1977, heavy precipitation (National Climate Data Center 2008) led to a rise in the water table and preceded the surfacing of some of the buried waste. Subsequent environmental sampling in homes adjacent to the waste site detected numerous volatile organic chemicals in basement air, suggesting a possible serious health threat via inhalation (Kim et al. 1982; NYSDOH 1981). In August 1978, the NYSDOH commissioner declared a health emergency at the Love Canal neighborhood (LC), and people nearest to the landfill were relocated (NYSDOH 1981). Shortly thereafter, President Carter declared a federal state of emergency, enabling the use of federal funds to aid in site remediation. In July 1980, Congress authorized funding for an additional emergency relocation of residents over a more extensive area (Fowlkes and Miller 1982; NYSDOH 1981). The area defined by these two evacuations (Figure 1) became known as the Emergency Declaration Area (EDA) [NYSDOH and U.S. Department of Health and Human Services (DHHS) 1986]. This man-made disaster encouraged the passage of the Comprehensive Environmental Response, Compensation, and Liability Act (1980) by the U.S. Congress, the legislation that authorized federal funding for Superfund remedial activities at hazardous waste sites nationwide.

Between 1978 and 1982, environmental sampling for chemicals in various media took place, and numerous health investigations of the former residents of the EDA were conducted. Two studies of potential relevance to the present study examined cytogenetic abnormalities (Heath et al. 1984; Picciano et al. 1980) among LC residents. Picciano et al. (1980) reported increased frequencies of cells with chromosome abnormalities, but this study suffered from lack of a control group, volunteer testing, small numbers, and lack of laboratory blinding. Heath et al. (1984), in a well-designed study, compared a small group of LC residents with residents in another Niagara County (NC) neighborhood not located near a waste site. Although these investigators found no elevations in chromosomal abnormalities among the LC residents compared with the controls, they cautioned that even with positive findings it would be difficult to predict later clinical illness in the individuals. The only study examining cancer incidence (Janerich et al. 1981) reported an elevated incidence of lung cancer for the census tract that contained the EDA but no elevations in the incidence of nine other cancers. Because of the sense of urgency during this time period, this study suffered from methodological limitations such as crude exposure estimates, an inadequate follow-up period for cancer outcomes, and lack of control for confounders, including smoking.

In 1996, the NYSDOH began a retrospective observational study of mortality, cancer incidence, and reproductive outcomes among the former LC residents to help address some of these issues. In 1998, an expert advisory committee convened to provide advice and guidance. A year later, three former LC residents were added to the committee to provide community input. The objectives of this article are to summarize the findings for cancer incidence by *a*) characterizing cancer incidence among the former residents from 1978 through 1996 compared with that of residents of NYS [exclusive of New York City (NYC)] and NC; and *b*) modeling cancer incidence with regard to measures of potential exposure to chemicals from the landfill.

## Materials and Methods

### Study area and population

The EDA consisted of 814 single-family homes as well as public housing apartments whose number is unknown because of the lack of real property information. The follow-up health study base population consisted of 6,181 persons. They lived in the Love Canal EDA some time between 1940 and June 1981, and they were interviewed by the NYSDOH in 1978–1982; subjects < 18 years of age were identified in a parental interview.

### Comparison populations

We identified two external populations for comparing cancer incidence rates with those of LC residents. We chose NYS excluding NYC because it was sufficiently large to provide stable cancer rates by year, age group, and sex. NC provided a comparison population similar to the LC cohort demographically, controlled for potential regional differences in the reporting of site-specific cancers, and allowed an attempt to control for possible local environmental sources of chemicals outside of the landfill.

### Tracing of the cohort

We traced the 6,181 members of the cohort beginning in 1996 and extending back in time to the date of their interview (1978–1982) to determine their current vital status and, if deceased, the date of death. We first submitted the names of all females to the New York State Vital Records (NYSVR) Marriage Registry to obtain information on possible name changes. We then matched all names associated with both male and female members of the cohort against the Social Security Death Index; to obtain current addresses of those not known to be dead, we used multiple sources that included the NYS Department of Motor Vehicles, Internet telephone directories, the U.S. Post Office Address Correction Service, and the NYSVR Death Registry. If those methods were not productive, we asked family members or former neighbors for information about the cohort member. Questionnaires were then mailed out to those with out-of-state addresses requesting detailed residential histories.

### Environmental sampling and exposure assessment

Environmental sampling by the U.S. EPA and the NYSDOH, begun in early spring of 1978, focused on indoor air, particularly in the basements and living spaces of homes closest to the buried wastes, because this was considered to be the most important route of exposure. Dermal contact was also a concern, so subsequent sampling was expanded to include soil, sediments, surface and storm sewer water, leachate (including non-aqueous-phase liquids), and some biota. Because the LC community was on a public water supply system, drinking water was not a potential route of exposure.

**Figure 1:**
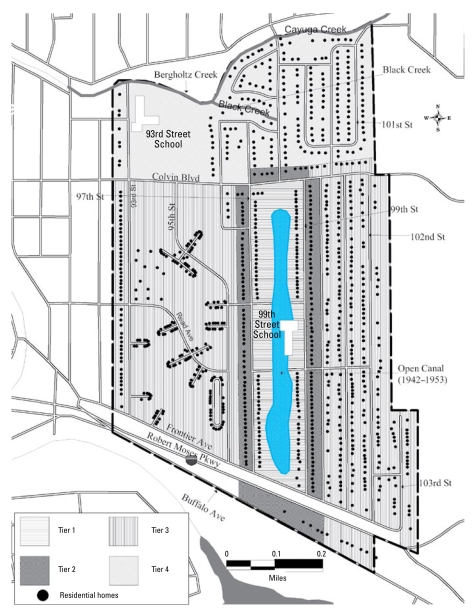
Love Canal Emergency Declaration Area (EDA). Reproduced from Gensburg et al. (2009) with permission from *Environmental Health Perspectives.*

Because environmental sampling was limited in scope and time frame, we conducted the internal comparisons among members of the LC cohort according to the potential for exposure of each cohort member to the landfill chemicals, based on qualitative factors. We constructed the primary exposure measures based on residential location and time period. We defined location by partitioning the EDA into four areas or tiers: tiers 1 and 2 were closest to the canal, and tiers 3 and 4 were farther away (Figure 1). We also identified two time periods of potential chemical exposure: 1942–1953 (the open period) and 1954 through the time of evacuation, ending in 1980 (the closed period). During the open period, the canal was uncovered, providing a greater chance for exposure through play, air transport and deposition, surface water run-off, and shallow groundwater transport. The landfill was covered in 1954, limiting direct access, although based on historical and environmental evidence the potential existed for continuing exposure (NYSDOH 1981). The resulting variables consisted of number of years of residence in one or more of four categories: *a*) open period, tiers 1 and 2; *b*) open period, tiers 3 and 4; *c*) closed period, tiers 1 and 2; and *d*) closed period, tiers 3 and 4. These variables were not mutually exclusive; many cohort members fell into more than one of the four categories. We constructed three additional exposure variables: childhood exposure, residence on a “hot spot” or swale, and attendance at the 99th Street School. We defined childhood exposure dichotomously (yes/no) as the additional potential for exposure among children growing up on Love Canal during the open and closed periods. Anecdotal evidence suggested that teenage boys swam in the water-filled trench during the years of active dumping; therefore, we considered males 13–18 years of age to be potentially exposed in childhood during the open period (1942–1953). After 1954, playing on the soil covering of the landfill was thought to be the main route of additional exposure for children; therefore, we considered children < 13 years of age who lived closest (tiers 1 and 2) during the closed period to be potentially exposed during childhood. We created a dichotomous variable to indicate whether the cohort member had lived in a residence built on one of the natural historic swales that may have served as conduits for the transport of chemicals. This variable also described residences where the 1978 sampling results indicated higher than expected levels of chemical contaminants in the soil, “hot spots” thought to be created from soil taken from the landfill. The third additional exposure variable was the number of years of attendance at the 99th Street School, an elementary school built adjacent to the landfill in 1954 and attended by students until 1978.

### Outcome assessment

We matched all persons in the LC cohort with the New York State Cancer Registry (NYSCR) using last and first name, month and year of birth, sex, Social Security number, and the soundex of first and last names. To assure a correct link, we visually reviewed all matches. The analyses included only incident cancers diagnosed from 1979 to 1996, because 1979 is the first year that the NYSCR was fully computerized. In an attempt to track former residents who had left NYS, we sent names to the eight states with registries to which the largest number of cohort members moved. Because of the low number of matches found and because gaps existed between the time these people moved to the state in question and out-of-state registries began operating, we included in the analyses only the portion of the follow-up period in which they were NYS residents.

The NYSCR also provided the cancer rates for the external comparison populations. We collected data by sex and age group from 1979 to 1996, employing the groupings used by the Centers for Disease Control and Prevention (CDC 2007). The year data groupings were 1979–1981, 1982–1986, 1987–1991, and 1992–1996. We coded diagnoses according to the *International Classification of Diseases, 9th Revision, Clinical Modification* (ICD-9-CM; DHHS 1989), in use from 1979 to 1998. Because small numbers prevented examination by most individual ICD-9 codes, we grouped sites by organ system.

### Potential confounders

To control for potential confounding of the association between cancer and exposure, we abstracted variables from the 1978–1982 interviews. This information included sex, date of birth, race, occupational narratives, history of cigarette smoking and alcohol consumption, and a general question on family history of cancer. We coded smoking and alcohol consumption variables as ever/never and family history as yes/no. Occupational histories included job titles, company names, and dates of employment. NYSDOH industrial hygienists reviewed this information to evaluate each job’s potential for exposure to carcinogens as high, medium, or low/no.

### Statistical analysis

To check for outliers and coding errors and to assess the properties of the data, we performed univariate analyses. We then generated descriptive statistics for all variables used in the analyses. We performed two major types of multivariable analyses: *a*) external comparisons, focusing on differences between the LC cohort and NYS and NC using standardized incidence ratios (SIRs), and *b*) internal comparisons using survival analysis methods, focusing on differences within the LC cohort according to the potential for exposure.

#### External comparisons

To compare the cancer incidence of the study group to NYS and NC, we calculated SIRs using the indirect method (Hennekens and Buring 1987). We computed person-years for the LC cohort as the difference from the date of interview to the cancer diagnosis, death, loss to follow-up, or end of the study period (31 December 1996). We computed point estimates for the SIRs as the ratio of observed to expected cases, and we calculated 95% confidence intervals (CIs) using the exact probabilities of the Poisson distribution. Expected numbers of cancers were based on age-, sex-, and year-specific population estimates and were adjusted by age group, year group, and sex. Results were also stratified by sex for NYS and NC.

#### Internal comparisons

We used the Cox proportional hazards model to statistically model the association between potential environmental exposure factors and cancer incidence among members of the LC cohort. This survival analysis focused on total cancers and three major categories of cancers: cancers of the digestive organs and peritoneum (ICD-9 codes 150–159), cancers of the respiratory and intrathoracic organs (ICD-9 codes 160–165), and cancers of the genitourinary tract (ICD-9 codes 179–189). We chose these categories because they contained sufficient numbers of cancers to allow for analyses. We also included two “environmentally sensitive” subgroups: cancers of the liver, rectum, and intrahepatic bile ducts (a subgroup of cancers of the digestive organs, ICD-9 codes 154–155), and bladder, kidney, and other urinary organs (a subgroup of genitourinary cancers, ICD-9 codes 188–189) because these cancers might be particularly affected by exposures to the chemicals in the landfill (liver, rectal, bladder, and kidney cancers), based on information from International Agency for Research on Cancer (IARC) monographs (IARC 1979), Agency for Toxic Substances and Disease Registry (ATSDR) toxicological profiles (ATSDR 2009), the National Toxicology Program (NTP) (Gold and Zeiger 1997), and the Carcinogenic Potency Database.

In addition to the seven exposure variables discussed above, we controlled for six variables obtained from the original 1978–1982 interviews as potential confounders in these analyses: age, sex, a history of smoking or alcohol consumption, a family history of cancer, and potential occupational exposure to carcinogens. For continuous variables, the hazard ratios (HRs) are per one-unit increments, and for the dichotomous variables, the HRs compare the two categories, with “no” being the reference. To test the proportionality assumption of the models, we included in the model the interactive terms for exposure variables of interest with survival time. Statistically significant terms were included in the model to correct for the non proportionality (Allison 1995), and Schoenfeld and Martingale residuals were plotted as an additional check to detect possible departures from the proportionality assumption (Hosmer and Lemeshow 1999).

Further details concerning the study methodology can be found elsewhere (Gensburg et al. 2009; NYSDOH 2006).

## Results

### Study population

The cohort for the external analysis included 5,052 men, women, and children either who never left New York (*n* = 4,461) or whose date of emigration was known (*n* = 591). Cohort members with the address information necessary for internal analysis numbered 5,007, and of these, 3,081 had complete interview data. Sociodemographic characteristics of these cohorts are presented in Table 1. The external cohort had more females (51.8%) than males, and most were white (94.3%). The median age at entry into the study for this cohort was 30 years, ranging from 0 to 94 years, and most (86.7%) resided in single-family dwellings during at least some of their time at LC, with 13.2% residing solely in public housing (data not shown).

We found similar proportions by sex and race for the internal analyses cohorts (Table 1). Because only persons ≥ 18 years of age were interviewed, the median ages at entry into the study for all interviewees and for those with complete interview information was 37 and 39 years, respectively, somewhat higher than for the full internal cohort (30 years; data not shown). The median number of years from date of first residence at LC until the end of follow-up was higher among interviewees with complete information (36 years) than for the full internal cohort (30 years), and the overall range was 1–55 years (data not shown). Most interviewees with complete information (62.8%) had occupational histories suggesting possible exposures to carcinogens, 71.2% had a history of smoking, 74.1% reported alcohol use, and 32.5% had a family history of cancer (Table 1).

### Exposure

Table 2 shows the time spent by the internal cohorts in each of the four time and tier exposure categories. For the full cohort (*n* = 5,007), median length of residence ranged from 1.6 years (range, 0.1–12 years) for those living in tiers 1 and 2 during the open period to 8.5 years (range, 0.1–27 years) for those living in tiers 3 and 4 during the closed period. The total number of years of residence at LC ranged from 0.1 to 39, with a median of 8.5 years. The median number of years attending the 99th Street School was 4.0 (range, 1–9 years), about 16% were exposed as a child to the chemicals deposited in the landfill, and 2.5% lived at some time on a hot spot or swale. The subset of interviewees with complete information, who were by definition older, were more likely to have been exposed in the open period and less likely to have attended the 99th Street School or to have been exposed as children.

### External comparisons

The 5,052-person cohort contributed 76,496 person-years to the analysis, with 304 incident cancers observed during the follow-up period. The description below refers to results using NYS as the standard population; the results relative to NC were generally similar (data not shown). Table 3 presents SIRs for cancer incidence among men and women, combined and separately, compared with NYS. We limited results to those sites for which at least one comparison *a*) had ≥ 5 observed cases and the SIRs exceeded 1.00, or *b*) had ≥ 10 observed cases. Many CIs are wide because of small numbers.

For total cancers, the SIR relative to NYS was 0.95 (95% CI, 0.83–1.05), with an SIR of 0.86 (95% CI, 0.72–1.01) among women and 1.02 (95% CI, 0.87–1.18) among men. Among organ systems, the most commonly observed cancer was genitourinary (SIRs = 0.81 for women, 1.09 for men). The sites with the greatest elevations in this group were bladder (SIR = 1.44; 95% CI, 0.91–2.16) and kidney (SIR = 1.48; 95% CI, 0.76–2.58). The second most common organ system cancer was of the digestive organs and peritoneum (SIR for both sexes combined = 1.03; 95% CI, 0.80–1.30). Site-specific SIRs in this category ranged from 1.08 (95% CI, 0.44–2.23) for cancer of the stomach (0.88 in women, 1.19 in men) to 1.28 (95% CI, 0.74–2.04) for cancer of the rectum (1.57 in women, 1.06 in men). Cancers of the respiratory and intrathoracic system was the third most frequent category (*n* = 62). Fifty-seven of the 62 cancers in this category were of the trachea, bronchus, and lung, with a combined SIR of 1.10 (0.94 for women, 1.20 for men).

### Internal comparisons

The cohort for survival analysis of cancer incidence included those who resided in NYS for the full follow-up period (*n* = 4,417) or for whom dates were available for when they left the state (*n* = 591). Of these 5,007 persons, 3,659 were interviewed, and 3,081 had complete interview data. Among this latter group, 268 cancers occurred. Table 4 shows a model containing relevant environmental, exposure, and background variables for these adults. None of the HRs for total cancers and the four time/tier exposure variables were substantially elevated. We found similar results for residence on a hot spot or swale, attending the 99th Street School, and childhood exposure. The results for the incidence of site-specific cancers were based on small numbers of observed cases and were not statistically significant in most cases.

The risks for genitourinary cancer (HR = 1.59; 95% CI, 0.39–6.55) and liver/rectal cancer (HR = 3.02; 95% CI, 0.39–23.18) were not statistically significantly elevated with respect to residing on a hot spot or swale. Among the interviewees with childhood exposure, genitourinary cancer (HR = 2.26; 95% CI, 0.42–12.20) and its subgroup, bladder and kidney cancer (HR = 17.35; 95% CI, 3.03–99.47), were elevated, based on two cases of bladder cancer. The HRs for age, male sex, and smoking were elevated for all cancer groupings, with statistically significant associations for age in all comparisons, male sex with most cancer groupings, and smoking with total cancers and respiratory cancers. We did not find statistically significant elevations for alcohol use, occupational exposure to carcinogens, or family history of cancer. Results for the 5,007 persons representing the whole internal cohort were not substantially different than those for the adults with complete interview information (data not shown).

Results for three additional definitions of exposure—total years of exposure, and continuous and categorical measures based on age and tier group—were mainly null (data not shown). Consistent with the above findings regarding childhood exposure, the HR for kidney/bladder cancers and years of residence as a child in tiers 1 or 2 was slightly elevated (HR = 1.13; 95% CI, 0.92–1.39) but not statistically significant. The numbers of exposed cases were too small in the cancer subgroups for analysis of the four age-group/tier variables as dichotomous indicator variables.

## Discussion

The overall cancer incidence of the LC cohort from 1979 to 1996 was similar to that of the general population of NYS and of NC, as was the cancer incidence of most major cancer site groupings. We found elevated SIRs relative to NYS for some cancer site groupings, including kidney and bladder for each sex, respiratory and stomach in men, and rectal cancer in women, although the 95% CIs for these groups included 1.00 because of small numbers. In the survival analysis, the numbers of cases for most cancer groups were too small to draw conclusions about the relationship between cancers and the environmental exposure variables. Consistent with the external analysis, however, we found elevated risks for childhood exposure and genitourinary cancers, and for its subcategory, bladder and kidney cancers, based on two cases of bladder cancer. The HRs for the four time-tier exposure variables were close to null for these cancers, as were the results of a sensitivity analyses that employed additional definitions of exposure based on age group and tier of residence. Cancer risks were elevated for established risk factors such as age, male sex, and smoking in the survival analyses for total cancers and cancer subgroups (Ezzati et al. 2003; DHHS 1982).

Other studies of cancer incidence or mortality associated with waste sites have yielded mixed results, and many studies were ecologic. The only other study of cancer incidence that includes the LC residents (Janerich et al. 1981) examined SIRs relative to NYS by age group and sex for each of 25 census tracts in the city of Niagara Falls, including the one containing the EDA. During 1966–1977, Janerich et al. (1981) found statistically significant elevations in respiratory cancers for the census tract containing the EDA (SIRs = 1.7 in men and 2.0 in women). However, they also found elevated respiratory cancers in other Niagara Falls census tracts and for the city as a whole. SIRs for urinary tract cancers for men and women in the census tract containing the EDA were 1.2 and 0.4, respectively, and were not statistically significant. Important limitations of that study included the short time from first exposure to diagnosis given the long latency periods required for most cancers, its ecologic nature, and the lack of information on smoking and other cancer risk factors. A second study examining lung cancer mortality in NC (Polednak and Janerich 1989) found no association with residence in census tracts containing toxic waste disposal sites.

Outside of NC, two ecologic studies conducted in populations living near landfills in Italy found statistically significant excesses of mortality from bladder cancer (Altavista et al. 2004; Minichilli et al. 2005); a large ecologic study in Great Britain found a slight excess risk of bladder cancer (SIR = 1.01; 95% CI, 1.00–1.02) in residents near landfill sites (Jarup et al. 2002); and another study (Griffith et al. 1989) found that among whites, the presence of National Priorities List sites in the United States was associated with mortality from cancers of the bladder, lung, stomach, and large intestine and rectum. A county-level ecologic study in New Jersey found associations between proximity to toxic waste sites and mortality from lung cancer (Najem and Molteni 1983), and gastrointestinal organ cancers (Najem et al. 1983), but not bladder cancer (Najem et al. 1984). Other similar studies did not find an elevation in total cancer incidence near hazardous waste sites (Baker et al. 1988; Najem et al. 1994). In summary, most other research was solely ecologic in nature and addressed cancer mortality rather than cancer incidence, but several studies of populations residing near sources of hazardous waste report results for bladder cancer that are consistent with those of the present study and consistent with toxicologic data from IARC, the ATSDR, and the NTP.

The present study has many notable strengths. The cohort is well defined, has known residential locations and dates, and almost all members (97%) were traceable, minimizing a potential source of selection bias. A wide range of total residential times existed (1 month to 39 years), and the cohort represents almost all areas of the Love Canal EDA. The length of the period between date of first exposure and end of follow-up ranged from 2 to 54 years (median, 35 years), which provided a sufficiently long latency period to allow time for most environmentally induced cancers to develop. We employed two complementary research designs: external and internal comparisons. The external comparisons allowed inclusion of all members of the LC cohort in the analysis, including those who were not interviewed. The internal comparisons allowed differential exposure assessment and control of potential confounders using interview data. In addition, we conducted a sensitivity analysis using more than one definition of exposure. Finally, the data on health outcomes came from the NYSCR, avoiding the potential biases associated with self-reported data.

Correspondingly, the study has important limitations. The study cohort may not have been representative of former residents of LC as a whole. By definition, the cohort used in the internal analysis was limited to adults with EDA residential information who completed interviews, and to their minor children. The unavailability of real estate records and the transience of a housing project sub population thwarted attempts to retrospectively create a complete list of everyone who had ever lived at LC from 1942 to 1980. To estimate the completeness of the cohort, we compared the size of our study population with census data by decade, beginning with 1960, because before that time the census reported population figures only at the county level. The EDA accounts for approximately half of its census tract, so we halved the census figures to estimate the number of persons living in the EDA for each decade. The results indicated that our cohort included about 95% of the population who lived there in the 1970s but only 60% of the population in the 1960s. This difference may reflect the facts that proportionately more of the older residents had died before 1978 and that it was more difficult to trace residents who lived at LC in the more distant past compared with more recent residents. Presumably the completeness of the cohort in the 1940s and 1950s would be lower yet. This differential completeness by time may have led to an underestimation of the cancer incidence in our cohort relative to that of the entire LC population, because cancer rates increase with age and because exposures may have been higher when Love Canal was open in the earlier years.

Additionally, this study excluded cancer diagnoses that occurred among cohort members before 1979 or after a cohort member emigrated from NYS, possibly biasing the results toward the null. Selection bias may have been introduced if the above factors were related to both exposure and outcome. We conducted a sensitivity analysis to attempt to assess the effect of missing cancer diagnoses among those who left the state before the end of the study period. Attributing the same rate of total cancers to those that left the state as were found in the full-time residents did not appreciably change the SIR compared with NYS.

Although statistical power was adequate for total cancer incidence for the cohort relative to the standard populations, power was low for all individual cancer sites. To increase power in the external analysis, in this study we also analyzed cancer end points by grouping those associated in the scientific literature with the greatest number of Love Canal chemicals, and by grouping them according to toxicologic end points. From these analyses, most SIRs were essentially 1.00, with only slight, non-statistically significant elevations for environmentally sensitive cancers or those related to known occupational hazards. A related weakness of this study was the increased likelihood of committing a type 1 error because of the large number of statistical comparisons made. Additionally, despite the long period between date of first exposure and end of follow-up, the cohort is still relatively young (median age in 1996, 49 years). As the cohort ages, more cases of cancer will likely develop, increasing statistical power to detect more consistent patterns between potential exposure and health effects.

Finally, there were no direct measurements of chemical contaminants in air, soil, and water before 1978. Therefore, the exposure variables used in this study were qualitative and aggregate, based on time, location, and age group. The lack of positive findings regarding most of these variables may be due, at least in part, to non differential misclassification, given the qualitative nature of the measures used. However, we are analyzing 373 serum samples collected in 1978 from members of the cohort for chemicals disposed of at Love Canal, and the results will help assess whether the qualitative variables used in this study correlate with body burden.

## Conclusion

In this study, our research objective was to assess the long-term health effects of residence at LC. Although the incidence of total cancers and most site-specific cancers among the LC residents from 1979 to 1996 were similar to that for the general population, we observed elevations of bladder and kidney cancers. We especially saw these elevations among residents potentially exposed as children. These findings are consistent with those of other studies, particularly with respect to bladder cancer. However, given the small number of cases and the other limitations of this study, the findings should be cautiously interpreted. Because many analyses were limited by small numbers of cancers and the study population is still relatively young, revisiting the cohort in the future could reveal patterns that are not yet apparent.

## Figures and Tables

**Table 1:** Summary of sociodemographic information on persons with known cancer status.

		Internal analysis cohorts (complete EDA address information)	
	External analysis cohort (*n* = 5,052)	Children and adults (*n* = 5,007)	Adults with complete interview information (*n* = 3,081)
Demographic information	[No. (%)]	[No. (%)]	[No. (%)]
Sex			
Male	2,437 (48.2)	2,419 (48.3)	1,459 (47.4)
Female	2,615 (51.8)	2,588 (51.7)	1,622 (52.6)
Race			
White	4,766 (94.3)	4,730 (94.5)	2,935 (95.3)
Nonwhite	272 (5.4)	266 (5.3)	246 (4.7)
Missing information	14 (0.3)	11 (0.2)	—
Occupationally exposed^a^ (365 missing)	—	—	1,934 (62.8)
Ever smoked (413 missing)	—	—	2,194 (71.2)
Ever used alcohol (421 missing)	—	—	2,283 (74.1)
Family history of cancer	—	—	1,002 (32.5)

a Possible occupational exposure to carcinogens.

**Table 2:** Exposure information: internal analysis.

	Children and adults (*n* = 5,007)		Adults with complete interview information (*n* = 3,081)	
Exposure Information	No. (%)^a^	Median years (range)	No. (%)^a^	Median years (range)
Open period, tier 1 or 2^b^	70 (1.4)	1.6 (0.1–12.0)	67 (2.2)	1.6 (0.1–12.0)
Open period, tier 3 or 4^b^	851 (17.0)	5.5 (0.1–12.0)	801 (26.0)	5.5 (0.1–12.0)
Closed period, tier 1 or 2	1,561 (31.2)	7.0 (0.1–25.0)	961 (31.2)	7.0 (0.1–25.0)
Closed period, tier 3 or 4	3,434 (68.6)	8.5 (0.1–27.0)	2,091 (67.9)	8.5 (0.1–27.0)
Attended 99th Street School	1,268 (25.3)	4.0 (1.0–9.0)	563 (18.3)	5.0 (1.0–9.0)
Total years of exposure	5,007 (100.0)	8.5 (0.1–39.0)	3,081 (100.0)	10.5 (0.2–39.0)
Exposed as a child	795 (15.9)	—	250 (8.1)	—
Residence on hot spot or swale	125 (2.5)	—	82 (2.7)	—

a Percentages for the first four exposure variables do not add up to 100.0 because the categories are not mutually exclusive.

b All residents living in the EDA during the “open” period were old enough to be interviewed in 1978.

**Table 3:** SIRs (year, age, and sex adjusted) for cancer among full- and part-time LC residents, compared with NYS.

		Males and females combined			Females			Males		
Cancer	ICD-9 code	Obs	SIR	95% CI	Obs	SIR	95% CI	Obs	SIR	95% CI
All cancers		304	0.95	0.83–1.05	142	0.86	0.72–1.01	162	1.02	0.87–1.18
Digestive organs and peritoneum	150–159	69	1.03	0.80–1.30	33	1.09	0.75–1.52	36	0.98	0.69–1.36
Stomach	151	7	1.08	0.44–2.23	2	0.88	0.11–3.20	5	1.19	0.39–2.78
Colon	153	26	0.88	0.58–1.30	14	0.96	0.52–1.61	12	0.81	0.42–1.42
Rectum	154	17	1.28	0.74–2.04	9	1.57	0.72–2.98	8	1.06	0.46–2.08
Respiratory and intrathoracic organs	160–165	62	1.07	0.82–1.37	19	0.87	0.53–1.37	43	1.18	0.86–1.59
Trachea, bronchus, and lung	162	57	1.10	0.83–1.42	19	0.94	0.57–1.47	38	1.20	0.85–1.64
Bone, connective tissue, skin, breast	170–175	50	0.80	0.59–1.06	47	0.84	0.61–1.11	3	0.49	0.10–1.43
Breast (female only)	174	42	0.82	0.59–1.10	42	0.82	0.59–1.10			
Genitourinary organs	179–189	82	0.78	0.77–1.22	26	0.81	0.53–1.18	56	1.09	0.82–1.41
Body of the uterus (female only)	182	5	0.50	0.16–1.16	5	0.50	0.16–1.16			
Ovary (female only)	183	9	1.14	0.52–2.16	9	1.14	0.52–2.16			
Prostate (male only)	185	29	0.91	0.61–1.30				29	0.91	0.61–1.30
Bladder	188	23	1.44	0.91–2.16	7	1.68	0.67–3.46	16	1.36	0.78–2.20
Kidney	189	12	1.48	0.76–2.58	4	1.30	0.35–3.32	8	1.59	0.69–3.13
Other and unspecified sites	190–199	18	0.94	0.56–1.49	10	0.98	0.47–1.81	8	0.90	0.39–1.76
Lymphatic and hematopoietic tissue	200–208	19	0.71	0.43–1.12	7	0.58	0.23–1.20	12	0.82	0.42–1.43

Obs, observed cases. Results are shown only for cancer subgroups that have > 10 observed cases or that have > 5 observed cases and SIRs > 1.00 for at least one comparison.

**Table 4:** Cox proportional hazards modeling for cancers in the interviewed cohort (*n* = 3,081).

	All sites (*n* = 268)		Respiratory and intrathoracic (*n* = 57)		Digestive organs and peritoneum (*n* = 64)		Liver and rectal (*n* = 19)		Genitourinary (*n* = 70)		Bladder and kidney (*n* = 32)	
Variable	n*	HR (95% CI)	n*	HR (95% CI)	n*	HR (95% CI)	n*	HR (95% CI)	n*	HR (95% CI)	n*	HR (95% CI)
Open period, tier 1 or 2 (years)^a^	8	0.95 (0.80–1.12)	2	1.04 (0.83–1.29)	0	—b	0	—b	4	1.01 (0.79–1.29)	3	0.93 (0.63–1.36)
Open period, tier 3 or 4 (years)^a^	104	1.00 (0.97–1.04)	20	0.95 (0.87–1.03)	27	1.03 (0.97–1.09)	9	1.07 (0.96–1.18)	37	1.04 (0.98–1.10)	17	1.04 (0.96–1.13)
Closed period, tier 1 or 2 (years)^a^	71	0.99 (0.97–1.01)	20	1.02 (0.98–1.06)	13	0.99 (0.94–1.03)	4	0.99 (0.93–1.07)	12	0.95 (0.90–1.00^–^)^c^	5	0.96 (0.89–1.03)
Closed period, tier 3 or 4 (years)^a^	177	0.99 (0.98–1.01)	32	1.00 (0.97–1.03)	50	1.01 (0.98–1.04)	11	0.99 (0.94–1.04)	49	0.98 (0.96–1.01)	24	1.00 (0.96–1.04)
Hot spot/swale (yes/no)	6	1.02 (0.45–2.31)	1	0.74 (0.10–5.40)	1	0.75 (0.10–5.44)	1	3.02 (0.39–23.18)	2	1.59 (0.39–6.55)	0	—b
Childhood exposure (yes/no)	5	0.99 (0.36–2.68)	0	—b	0	—b	0	—b	2	2.26 (0.42–12.20)	2	17.35 (3.03–99.47)
Attended 99th Street School (years)^a^	10	0.95 (0.82–1.10)	1	0.85 (0.54–1.36)	1	0.85 (0.55–1.30)	0	—b	4	1.04 (0.81–1.34)	1	0.49 (0.14–1.70)
Age (years)		1.07 (1.06–1.08)		1.09 (1.07–1.12)		1.08 (1.06–1.10)		1.10 (1.06–1.15)		1.09 (1.06–1.11)		1.09 (1.05–1.12)
Male (yes/no)		1.53 (1.12–2.08)		2.20 (1.08–4.52)		2.12 (1.09–4.15)		2.02 (0.57–7.13)		2.56 (1.35–4.85)		3.42 (1.29–9.09)
Ever smoked (yes/no)		2.08 (1.50–2.90)		6.54 (2.00–21.42)		1.67 (0.89–3.13)		2.21 (0.60–8.17)		1.69 (0.90–3.18)		1.38 (0.57–3.35)
Alcohol consumption (yes/no)		0.90 (0.68–1.18)		1.09 (0.59–2.01)		0.77 (0.45–1.32)		2.23 (0.68–7.30)		0.94 (0.54–1.64)		0.63 (0.29–1.34)
Potential occupational exposure to carcinogens (yes/no)		0.84 (0.61–1.16)		1.05 (0.48–2.31)		0.60 (0.31–1.16)		0.53 (0.15–1.82)		1.05 (0.53–2.08)		1.26 (0.42–3.72)
Family history of cancer (yes/no)		1.28 (1.00–1.64)		1.08 (0.62–1.88)		1.16 (0.69–1.94)		0.85 (0.32–2.26)		1.33 (0.82–2.16)		0.84 (0.39–1.83)

n* , number of cases in each exposure category.

a HRs calculated per 1-year increments.

b HR not calculable because of zero cells.

c 1.00^−^ indicates a number slightly less than 1.00.
